# Topographic Organization of Inferior Olive Projections to the Zebrin II Stripes in the Pigeon Cerebellar Uvula

**DOI:** 10.3389/fnana.2018.00018

**Published:** 2018-03-15

**Authors:** Iulia Craciun, Cristián Gutiérrez-Ibáñez, Jeremy R. Corfield, Peter L. Hurd, Douglas R. Wylie

**Affiliations:** ^1^Neuroscience and Mental Health Institute, University of Alberta, Edmonton, AB, Canada; ^2^Department of Biological Sciences, Salisbury University, Salisbury, MD, United States

**Keywords:** cerebellum, inferior olive, uvula, climbing fiber, optic flow, zebrin II, aldolase C

## Abstract

This study was aimed at mapping the organization of the projections from the inferior olive (IO) to the ventral uvula in pigeons. The uvula is part of the vestibulocerebellum (VbC), which is involved in the processing of optic flow resulting from self-motion. As in other areas of the cerebellum, the uvula is organized into sagittal zones, which is apparent with respect to afferent inputs, the projection patterns of Purkinje cell (PC) efferents, the response properties of PCs and the expression of molecular markers such as zebrin II (ZII). ZII is heterogeneously expressed such that there are sagittal stripes of PCs with high ZII expression (ZII+), alternating with sagittal stripes of PCs with little to no ZII expression (ZII−). We have previously demonstrated that a ZII+/− stripe pair in the uvula constitutes a functional unit, insofar as the complex spike activity (CSA) of all PCs within a ZII+/− stripe pair respond to the same type of optic flow stimuli. In the present study we sought to map the climbing fiber (CF) inputs from the IO to the ZII+ and ZII− stripes in the uvula. We injected fluorescent Cholera Toxin B (CTB) of different colors (red and green) into ZII+ and ZII− bands of functional stripe pair. Injections in the ZII+ and ZII− bands resulted in retrograde labeling of spatially separate, but adjacent regions in the IO. Thus, although a ZII+/− stripe pair represents a functional unit in the pigeon uvula, CF inputs to the ZII+ and ZII− stripes of a unit arise from separate regions of the IO.

## Introduction

**Figure 1:**
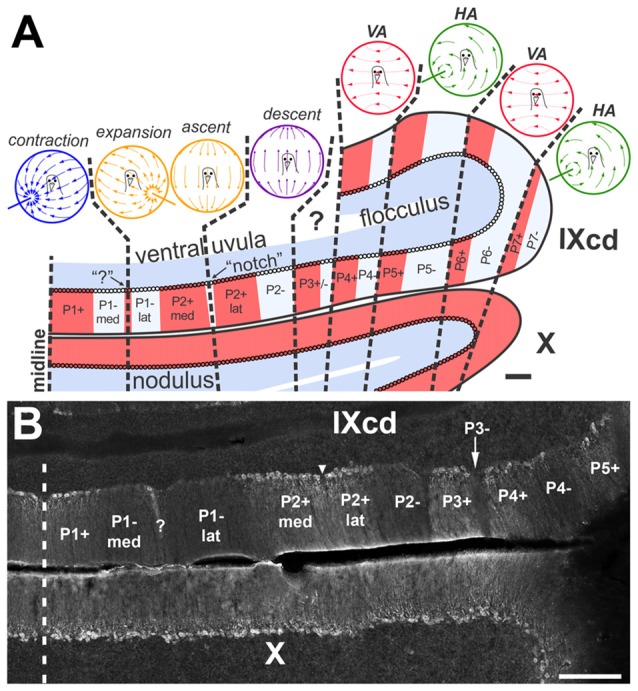
**(A)** shows the optic flow zones and zebrin II (ZII) pattern in the pigeon vestibulocerebellum (VbC). A coronal section through the VbC (folia IXcd and X) is depicted. The alternating ZII+ (red) and ZII− (pale blue) stripes are numbered from P1+/− most medially to P7+/− laterally, with ZII+ and ZII– stripes alternating (Adapted from Pakan et al., 2014 with permission). **(B)** is a photomicrograph of ZII immunoreactivity through a caudal section of IXcd showing stripes P1+ through P5+. P1− is bisected by a satellite ZII+ band designated “?”, dividing the stripe into medial and lateral halves (P1−med; P1−lat). P2+ is divided by a ZII– “notch”, indicated by the inverted triangle, dividing P2+ into medial and lateral halves (P2+med; P2+lat) (Wylie et al., 2007). In the uvula, the optic flow zones from medial to lateral are as follows: contraction zone (P1+/P1– med); expansion/ascent zone (P1–lat/P2+med); descent zone (P2+lat/P2–) (Graham and Wylie, 2012). Purkinje cells (PCs) in the flocculus zones respond to optic flow resulting from rotation about either the vertical axis (VA) or an horizontal axis (HA). There are two VA zones, spanning P4+/− and P6+/−, inderdigitated with two HA zones, spanning P5+/− and P7+/− (Pakan et al., 2011). PCs in the P3+/− stripe do not respond to optic flow stimuli and their function is unknown (Graham and Wylie, 2012). The sagittal organization of the optic flow zones extends into the nodulus (folia X), although all PCs are ZII+ (Pakan et al., 2007, 2011, 2014; Pakan and Wylie, 2008; Graham and Wylie, 2012). Scale bars = 200 μm in **(A)**, 300 μm in **(B)**.

The cerebellum exhibits an organization defined by sagittal zones (Voogd and Bigaré, 1980), which is evident with respect to the distribution of afferent input from climbing fibers (CFs) and mossy fibers, the projection patterns of efferent Purkinje cell (PC) outputs, as well as from the response properties and synchronous firing of PCs (Llinas and Sasaki, 1989; De Zeeuw et al., 1994; Voogd and Glickstein, 1998; Wu et al., 1999; Ruigrok, 2003; Apps and Garwicz, 2005; Pakan and Wylie, 2008; Pakan et al., 2011, 2014; Graham and Wylie, 2012). Additionally, several molecular markers exhibit parasagittal expression in the cerebellum. The most extensively studied in this regard is zebrin II (ZII). The ZII antibody recognizes the 36-kDa metabolic isoenzyme aldolase C and is expressed exclusively by PCs (Brochu et al., 1990; Ahn et al., 1994; Hawkes and Herrup, 1995). ZII is expressed heterogeneously such that bands of high ZII expression (ZII+) are interdigitated with bands of little to no ZII expression (ZII−; Figure 1). ZII stripes are seen in several mammalian (Leclerc et al., 1992; Ozol et al., 1999; Armstrong and Hawkes, 2000; Sanchez et al., 2002; Marzban et al., 2003, 2015; Sillitoe et al., 2003a,b; Marzban and Hawkes, 2011) and avian species (Pakan et al., 2007; Iwaniuk et al., 2009; Marzban et al., 2010; Corfield et al., 2015, 2016; Vibulyaseck et al., 2015), as well as in one genus of lizards (Wylie et al., 2016). The prevalence of ZII stripes across various species suggests that the role for ZII is highly conserved, and is likely crucial to cerebellar function. Several studies have examined the relationship between ZII stripes and the aforementioned aspects of sagittal cerebellar organization (Gravel and Hawkes, 1990; Hawkes and Gravel, 1991; Matsushita et al., 1991; Akintunde and Eisenman, 1994; Chockkan and Hawkes, 1994; Ji and Hawkes, 1994; Voogd et al., 2003; Sugihara and Shinoda, 2004, 2007; Voogd and Ruigrok, 2004; Wadiche and Jahr, 2005; Gao et al., 2006; Pijpers et al., 2006; Sugihara and Quy, 2007; Sugihara et al., 2007; Ruigrok et al., 2008; Mostofi et al., 2010; Paukert et al., 2010; Sugihara, 2011; Zhou et al., 2014).

In the pigeon vestibulocerebellum (VbC), we have shown that the ZII stripes coincide with functional properties of PCs and afferent inputs (Pakan and Wylie, 2008; Pakan et al., 2010, 2011, 2014; Graham and Wylie, 2012; Wylie et al., 2013, 2017). The avian VbC, which consists of folia IXcd and X, contains PCs whose complex spike activity (CSA) are responsive to optic flow patterns resulting from self-motion (Wylie and Frost, 1991). Optic flow results from visual motion occurring across the entire retina, and is important for many behaviors such as navigating, controlling posture and locomotion, and perceiving self-motion. The pathways that are involved in optic flow processing originate from retino-recipient nuclei in the pretectum and the accessory optic system and reach the medial column of the inferior olive (mcIO), which innervates the vestibulocerebellar optic flow zones (for review, see Wylie, 2013). The functional organization of the optic flow zones is shown in Figure 1 (Pakan et al., 2011; Graham and Wylie, 2012; Wylie, 2013). There are seven ZII+/− stripe pairs in IXcd (P1+/− to P7+/−), but all PCs in X are ZII+ (Pakan et al., 2007). Nonetheless, the optic flow zones span both folia. In the lateral aspect of the VbC, the flocculus, PC CSA responds best to optic flow resulting from self-rotation about either the vertical axis (VA), or an horizontal axis (HA; Wylie and Frost, 1993). The flocculus contains two VA zones interdigitated with two HA zones. The VA zones correspond to ZII stripes P4+/– and P6+/−, whereas the HA zones correspond to the P5+/− and P7+/− stripes (Pakan et al., 2011). In the medial VbC, the ventral uvula (IXcd) and nodulus (X), PC CSA responds to patterns of optic flow resulting from self-translation, while the dorsal uvula is not modulated by optic flow stimuli (Wylie et al., 1993, 1998). Within the ventral uvula and nodulus, there are four types of neurons organized into three sagittal zones (Graham and Wylie, 2012). In the most medial zone, which spans P1+ and the medial half of P1− (P1−med), PCs respond to “contraction” optic flow; i.e., optic flow resulting from backward translation. Adjacent to this is a zone that spans the lateral half of the P1− stripe (P1−lat) and the medial half of P2+ (P2+med). The P1− stripe is bisected by a thin ZII+ stripe 1–3 PCs in width (see “?” in Figure 1B). Similarly the P2+ stripe is bisected by a thin a notch that contains no PCs (see inverted triangle in Figure 1B; Pakan et al., 2007). Intermingled in this zone are two types of optic flow PCs: either “expansion” optic flow, or optic flow resulting from “ascent” (i.e., upward translation). Finally, lateral to this and spanning the lateral half of P2+ (P2+lat) and the P2− stripe, is a zone containing neurons responsive to optic flow resulting from “descent”. Previously we have shown that the inputs to the ZII+ and ZII− stipes in the flocculus (i.e., stripes P4+/− to P6+/−) receive CF input from separate, but adjacent areas of the inferior olive (IO; Wylie et al., 2017). The ZII+ stipes within the VA zones (P4+ and P6+) receive input from the caudal-most portion of the mcIO while the ZII− stripes of the VA zones (P4− and P6−) are innervated by a slightly more rostral region of the mcIO. Similarly, the ZII+ stripes of the HA zones are innervated by a more caudal region of the mcIO than the ZII− stripes of the HA zone, which are innervated by the rostral-most mcIO (Wylie et al., 2017). Thus, although a ZII+/− stripe pair represents a functional unit in the flocculus whereby all PCs respond to the same pattern of optic flow (either VA or HA), the ZII+ and ZII− stripes receive optic flow from different areas of the IO. In the present study we sought to determine if the same scheme holds true for the ventral uvula.

## Materials and Methods

### Surgical Procedure and Tracer Injection

The methods used adhere to the guidelines established by the Canadian Council on Animal Care and were approved by the Biosciences Animal Care and Use Committee at the University of Alberta. Twelve rock pigeons (*Coulmba livia*) of either sex, obtained from a local supplier, were anesthetized with an intramuscular injection of a ketamine (65 mg/kg) and xylazine (8 mg/kg) cocktail. Supplemental doses were administered as necessary. Animals were placed into a stereotaxic device, and their heads stabilized with pigeon ear bars and a beak bar adapter so that the orientation of their skull conformed to the atlas of Karten and Hodos (1967). Sufficient bone and dura were removed to allow access to the uvula with vertical penetrations. To localize the desired ZII stripe for injection of retrograde tracer (CTB; Cholera Toxin Subunit B), responses of PC CSA to visual stimuli were obtained using extracellular recording techniques. Glass micropipettes with tip diameters of 3–5 μm, filled with 2 M NaCl were advanced through the brain into the uvula using an hydraulic microdrive (Frederick Haer). Extracellular signals were then amplified, filtered, and fed through a window discriminator to record isolated PC CSA. The signal from the widow discriminator was fed to an oscilloscope and an audio monitor. The visual stimulation procedure has been described in detail elsewhere (Graham and Wylie, 2012). Briefly, the optic flow preference of the CSA was qualitatively determined by moving a large (90 × 90°) handheld visual stimulus, consisting of black bars, wavy lines and dots on a white background, in various directions throughout the visual field. Several recording tracts were made such that we could obtain a rough map of the locations of the optic flow zones in the uvula, and thus the presumed locations of the ZII stripes in the uvula (Figure 1A). Once the desired ZII stripe was located, the recording electrode was replaced with a micropipette (tip diameter 20–30 μm) filled with a retrograde tracer: fluorescent Cholera Toxin Subunit B (CTB; 1% in 0.1 M PB); either CTB-Alexa Fluor 488 (green) or 594 (red) conjugate (ThermoFisher Scientific). CSA was again recorded with the injection micropipettes to ensure injection in the desired ZII stripe. CTB was iontophoresed for 7 min, at +5 μA, for 7 s on, 7 s off. In some cases, we repeated this procedure to inject the adjacent ZII stripe using the CTB opposite in color to that used in the first injection.

### Post-Surgery, Recovery and Perfusion

After surgery, the craniotomy was filled with bone wax, and the wound was sutured. An intramuscular injection of buprenorphine (0.012 mg/kg) was given as an analgesic. Animals were left to recover for 5 days, allowing the retrograde tracer time to travel to the mcIO. After the recovery period, the animals were deeply anesthetized with sodium pentobarbital (100 mg/kg) and a transcardial perfusion with phosphate buffered saline (PBS; 0.9% NaCl, 0.1 M phosphate buffer) followed by 4% paraformaldehyde in 0.1 M PBS (pH 7.4) was performed. The brains were extracted from the skull and immersed in paraformaldehyde for between 2–7 days at 4°C. The brains were then placed in 30% sucrose in 0.1 M phosphate buffer until they sank (48–72 h). They were subsequently embedded in gelatin and cryoprotected in 30% sucrose in 0.1 M PBS overnight. The gelatin-embedded brains were frozen, and sliced into 40 μm thick coronal sections using a sliding microtome. Sections were collected in three series through the rostro-caudal extent of the cerebellum and the brainstem. Sections were stored in 0.1 M PBS (pH 7.4) between sectioning and immunohistochemistry.

### Immunohistochemistry

ZII immunohistochemistry was used to verify the locations of CTB injections. The sections containing injection sites in the uvula were processed for ZII expression. Tissue was rinsed thoroughly with 0.1 M PBS, then blocked with 10% normal donkey serum (Jackson Immunoresearch Laboratories, West Grove, PA, USA) and 0.4% Triton X-100 in PBS for 1 h to block non-specific binding of antibody. Tissue was then incubated in PBS containing 0.1% Triton X-100, and the primary antibody, anti-ZII/aldolase C (1:1000, goat-polyclonal, sc-12065, Santa Cruz Biotechnologies, Santa Cruz, CA, USA) for 120 h at 4°C. After primary antibody incubation, sections were rinsed in 0.1 M PBS and incubated in a fluorescent secondary; either AlexaFluor 594 or 488 anti-goat antibody (1:200, Jackson Immunoresearch Laboratories, West Grove, PA, USA) in PBS, 2.5% normal donkey serum, and 0.4% Triton X-100 for 3 h at room temperature. In cases of a single injection, the secondary antibody used was the color opposite to that of the CTB (i.e., red CTB, green secondary; green CTB, red secondary). In cases where both CTB colors were injected, the red and green secondary was used on alternate sections. The tissue was then rinsed five times in 0.1 M PBS and mounted onto gelatinized slides for viewing.

### Microscopy and Image Analysis

Sections were viewed with a compound light microscope (Leica DMRE, Richmond Hill, ON, USA) equipped with TX2 (red) and L5 (green) fluorescence filters. Images were captured with a Retiga EXi *FAST* Cooled Mono 12-bit camera (QImaging, Burnaby, BC, USA) using OpenLAB imaging software (Improvision, Lexington, MA, USA). Adobe Photoshop (San Jose, CA, USA) was used to adjust contrast and brightness.

**Figure 2:**
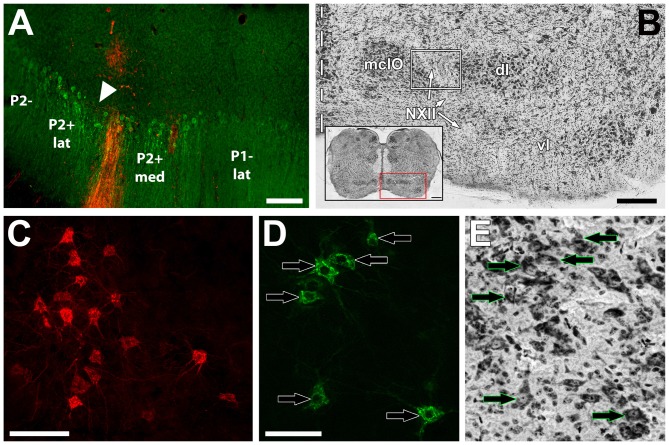
**(A)** shows an example of a Cholera Toxin B (CTB) injection site (red) in a section immunoprocessed for ZII (green; i.e., Alexafluor 488 scndry antibody). The injection was in the P2+med stripe of the uvula. The triangle indicates the ZII− notch separating the P2+med and P2+lat. **(B)** shows a thionine-stained section through the inferior olive (IO) showing the three subdivisions: medial column of the inferior olive (mcIO), ventral lamella (vl) and dorsal lamella (dl). The course of the twelfth nerve (NXII) is through the lateral part of mcIO. The white box indicates the general region where retrograde labeled cells were found from injections in the uvula. The inset shows a coronal cross section through the medulla and the red box indicates the region where the olive is found. **(C,D)** respectively, show retrograde labeled cells from red and green CTB injections. **(E)** shows the same section in **(D)** subsequently stained with thionine. Arrows in **(D,E)** indicate corresponding neurons. Scale bars = 200 μm in **(A)**; 250 μm in **(B)**; 500 μm in **(B)** inset; 100 μm in **(C)**; 50 μm in **(E)**.

**Figure 3:**
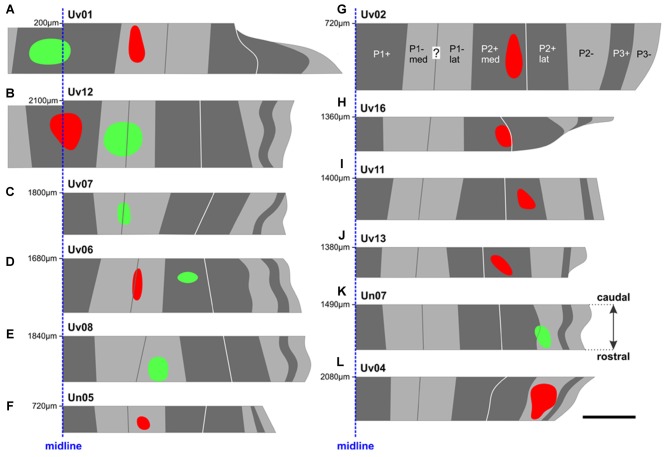
The locations of all CTB injection sites in the ventral uvula were reconstructed from serial coronal sections. The color of the injection site indicates the color of CTB injected (red or green). Injection sites **(A–L)** are shown from most medial in **(A,B)** (P1+) to most lateral in **(K,L)** (P2–). For each case, the distance (in μm) of the caudal-most section (i.e., top) from the posterior pole of IXcd is indicated. For example, the injections in case Uv01 **(A)** were located about 2 mm posterior to those in case Uv12 **(B)**. The midline is represented as a dashed blue line. See Table 1 for a detailed account of each injection site. Scale bar = 500 μm.

Injection site location was confirmed using ZII immunohistochemistry and was mapped out to determine the location of tracer within the stripes (Figures 2A, 3). For two out of three series, we measured the distance from the midline to the borders of each ZII stripe as well as the distance from the midline to the injection site borders. This allowed us to plot the injection site map as seen in Figure 3. Using Adobe Photoshop, we calculated the percentage of tracer in each ZII stripe for the cases where the injection spanned different ZII stripe signatures (Table 1). To do so, we selected portions of the injection in different stripes from our reconstruction (Figure 3) and used a measurement function to provide dimensions for the area selected.

**Table 1:** Summary of uvular injections by zebrin II (ZII) stripe.

Stripe case	P1+	P1-med	P1-lat	P2+med	P2+lat	P2-
Uv01 grn	G* 61 *(100%)*					
Uv01 red		**R 44** *(100%)*				
Uv12 red	R* 95 *(100%)*					
Uv12 grn		**G 118** *(63%)*	*(37%)*			
Uv07		*(43%)*	**G 60** *(57%)*			
Uv06 red		*(16%)*	**R 61** *(84%)*			
Uv06 grn				**G 26** *(100%)*		
Uv08			**G 61** *(100%)*			
Un05			**R 19** *(100%)*			
Uv02				**R 29** *(100%)*		
Uv16				**R 61** *(99%)*		
Uv11					**R 57** *(100%)*	
Uv13					**R 88** *(100%)*	
Un07					*(16%)*	**G 88** *(84%)*
Uv04						**R 99** *(100%)*

R and G indicate injections of red and green CTB, respectively (*indicates bilateral labeling). The number of retrogradely labeled cells in the medial column of the inferior olive (mcIO) is indicated in bold. Beside in parentheses, the proportion (in %) of the area of the injection site in each stripe is indicated.

Images were obtained of all sections containing retrogradely labeled IO neurons (Figures 2C,D). Subsequently, for one of the three series, the sections were Nissl-stained with thionine to allow visualization of the IO and other nuclei in the brainstem (Figures 2B,E). The thionine-stained sections were imaged, and the respective fluorescent images were overlaid onto the thionine images using Adobe Photoshop, to determine the location of the fluorescing cells within the IO (Figures 2D,E). For comparison of the locations of retrograde labeled mcIO cells across cases, sections were overlaid and aligned using several landmarks including: the caudal and rostral tips of the IO, the midline of the brain, the separation point of the ventral and dorsal lamella of the IO, and the course of the hypoglossal nerve (NXII).

## Results

The results are based on 12 animals in which we injected red and/or green fluorescent CTB into either a positive or negative stripe of a functional ZII stripe pair. A typical injection site is shown in Figure 2A. The location of each injection in relation to the ZII stripes was reconstructed from serial sections and plotted in Figure 3. Note that some of these injections extended across the border of a ZII stripe. Using *Adobe Photoshop*, we were able to measure the proportion (in percent) of the injection in the targeted ZII stripe as well as the spread into adjacent stripes. For each case, this is indicated in Table 1, along with the total number of retrograde labeled cells in the mcIO. In nine animals, a single CTB injection (red or green) was considered, whereas in three animals, there were two injections of different colors.

**Figure 4:**
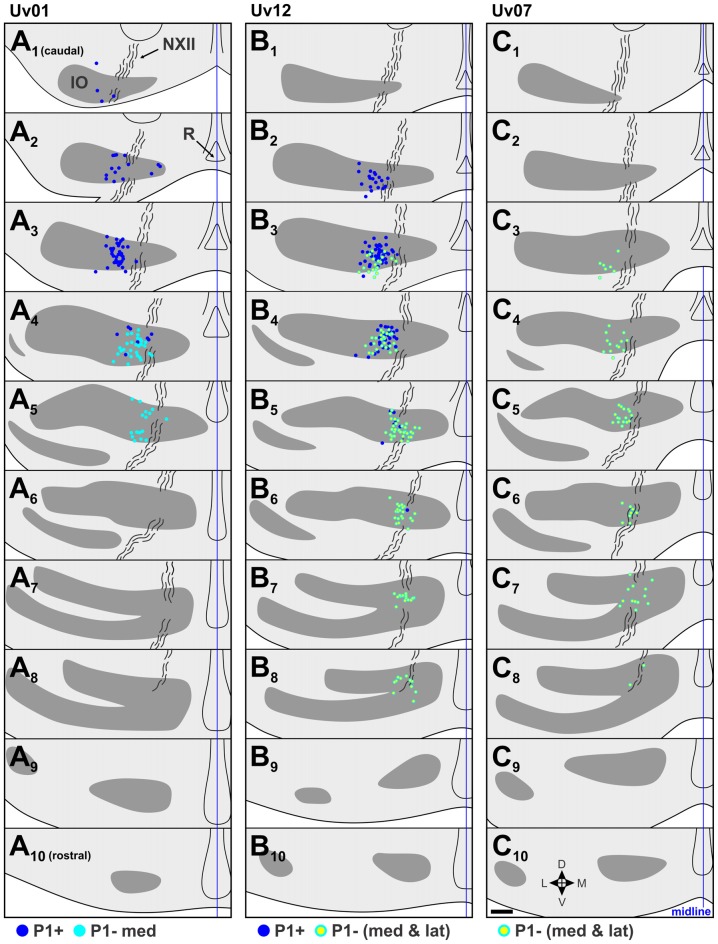
Retrograde labeling in the IO from selected P1+ and P1– injections in the contraction zone of the ventral uvula (see Figure 3). **(A)**, **(B)** and **(C)** respectively, show the retrograde labeling from cases Uv01, Uv12 and Uv07. For each, 10 coronal sections through the IO from 1 series (of 3) are shown, from caudal (top, **1**) to rostral (bottom, **10**), 120 μm apart. For each section, labeled cells from that section are shown, as well as labeled cells from adjacent sections: i.e., retrograde labeling is superimposed from three consecutive 40 μm sections. The IO is represented by gray shading, and the curvy black lines represent the course of the hypoglossal nerve (NXII). Dark blue dots represent retrograde labeled cells from the P1+ injections **(A,B)**, light blue dots represent those from the P1–med injection **(A)**, and light blue dots with yellow centers represent cells labeled from the injections that included both the medial and lateral halves of P1− (P1−med and P1−lat) **(B,C)**. d, dorsal; v, ventral; m, medial; l, lateral; R, raphe nucleus. Scale bar = 200 μm.

**Figure 5:**
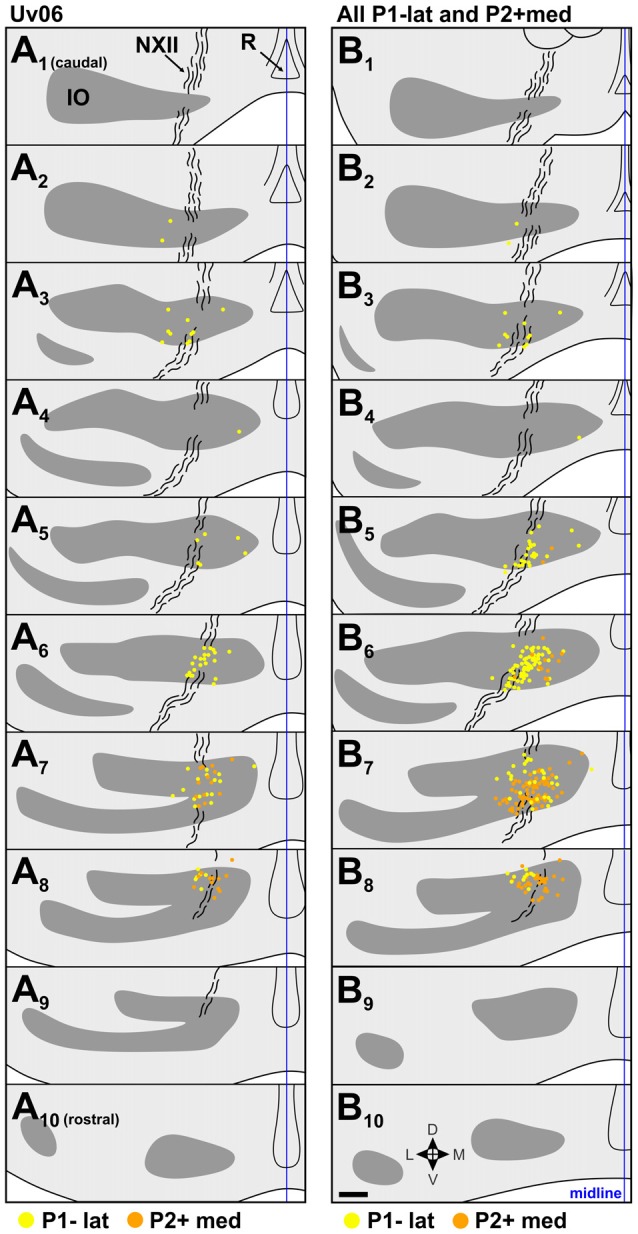
Retrograde IO labeling from injections in the expansion/ascent optic flow zone. **(A)** shows labeled cells from case Uv06, and **(B)** shows labeling from all injections in P1−lat and P2+med collapsed onto an idealized series (cases Uv06, Uv08, Un05, Uv02, Uv16; see Figures 3D–H and Table 1). The locations of labeled cells from injections in P1−lat and P2+med are represented by yellow and orange dots, respectively. See caption to Figure 4 for additional details. Scale bar = 200 μm.

**Figure 6:**
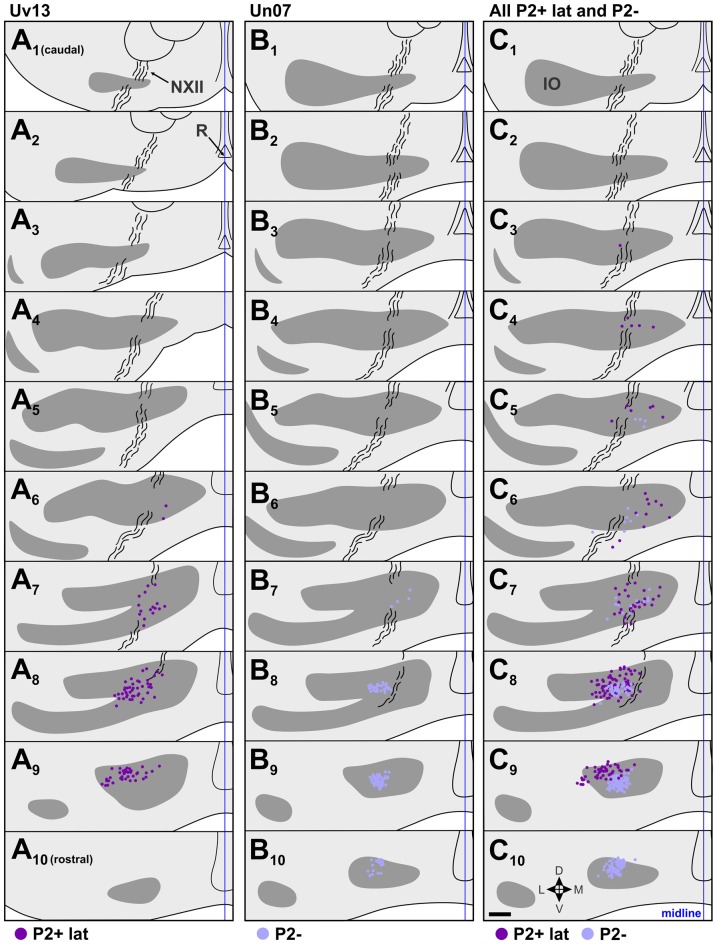
Retrograde IO labeling from injections in the descent optic flow zone. **(A,B)** show the locations of retrogradely labeled cells in the IO from cases Uv13 and Un07, respectively. **(C)** shows the labeling from all injections in P2+lat and P2− collapsed onto an idealized series (cases Uv11, Uv13, Un07, Uv04; see Figures 3I–L and Table 1). The locations of labeled cells from injections in P2+lat and P2− are represented by dark purple and light purple dots, respectively. See caption to Figure 4 for additional details. Scale bar = 200 μm.

The pigeon IO can be divided into three regions; the ventral lamella (vl), the dorsal lamella (dl) and the medial column (mcIO; Arends and Voogd, 1989; Figure 2B). From all injections, retrograde labeling was found in the lateral margin of contralateral mcIO often within the genu of the XII nerve (Lau et al., 1998; Crowder et al., 2000; Figure 2B). This region is immediately lateral to that which provides CF input to stripes P4+/− to P7+/− in the flocculus (Wylie et al., 1999, 2017). The average number of retrograde labeled mcIO cells from each injection was 64.5 ± 7.4 (mean ± SEM). Photomicrographs of retrograde labeled cells in the mcIO are shown in Figures 2C,D. Retrograde labeled olivary cells were confined to separate regions depending on the injection site location (Figures 4–6). For each optic flow zone, we describe the distribution of retrograde labeled cells in terms of their rostro-caudal relationship. Broadly speaking however, labeled cells in the rostral regions of the mcIO are generally located ventrally, while cells in caudal mcIO regions are distributed more dorsally.

The illustrated sections in Figures 4–6 represent every third collected section, and are 120 μm apart. In each section, the locations of retrograde labeled cells from adjacent sections are superimposed. In other words, in each section, the retrograde labeled cells in that section are shown, plus those from the sections 40 μm caudal and 40 μm rostral.

### Contraction Zone; P1+ vs. P1–med

There were two injections in P1+ (Uv01, Uv12; Figures 3A,B), both of which crossed the midline and resulted in bilateral labeling in mcIO. There was one injection confined to P1−med (Uv01) and two other cases where P1−med was targeted, but the injection was clearly spread into P1−lat (Uv12, Uv07; Figures 3A–C, see also Table 1). The resultant retrograde labeling from these cases is shown in Figure 4. In Figures 4A,B (Uv01, Uv12) the locations of retrograde labeled IO neurons from the P1+ injections are shown as dark blue. As these injections were bilateral, the labeling in the right IO has been superimposed on the left IO. From both cases, most of the labeling was clustered caudally, in sections A_2_/B_2,_ A_3_/B_3_ and B_4._ The retrograde labeling from the P1−med injection (Uv01), shown as light blue, was found more rostrally, mainly in sections A_4_ and A_5_. Labeling from the two injections that spanned the P1−med and P1−lat stripes (Uv12, Uv07; light blue dots with yellow centers) was also found at this rostro-caudal level (B_4,5_/C_4,5_), but also spread more rostrally (B_6–8_/C_6,7_). From these data, we conclude that the IO cells projecting to the P1−med stripe are immediately rostral to those projecting to the P1+ stripe.

### Expansion/Ascent Zone; P1–lat vs. P2+med

The location of injection sites in the expansion/ascent zone are shown in Figures 3D–H. Case Uv06 included injections in both the P1−lat and P2+med, although the P1−lat injection spread slightly to the P1−med stripe (Table 1). Two other cases included a single injection in P1–lat (Uv08, Un05), and two others targeted the P2+med stripe (Uv02, Uv16). There was minimal spread to the P2+lat stripe in case Uv16. The retrograde labeling from case Uv06 is shown in Figure 5A, where those cells labeled from the P1−lat and P2+med injections are shown as yellow and orange dots, respectively. Labeling from the P1−lat injection was concentrated in the sections A_6_ and A_7_ (this is consistent with the labeling in Figures 4B,C which spanned the P1−med and P1−lat stripes). We suspect the sparse labeling found caudally may be due to spread of the injection into the P1−med stripe. Cells labeled from the P2+ injection were concentrated more rostrally in the sections A_7_ and A_8_. In Figure 5B, the labeling from all 5 cases involving injections in P1−lat and P2+med has been collapsed onto a single idealized series. Although there is some overlap in section B_7_, the labeling from the P1−lat injections is clearly caudal to that from the P2+med injections.

### Descent Zone; P2+lat vs. P2–

Figures 3I–L shows the location of injection sites in the descent zone. Two injections were aimed at the P2+lat stripe (cases Uv11, Uv13), and two were aimed at the P2− stripe (cases Uv04, Un07). The injection in Un07 was the only one of these that showed spread to an adjacent stripe (84% in P2−, 16% in P2+lat; Table 1). Retrograde labeling (dark purple dots) from case Uv13 (P2+lat) is shown in Figure 6A, where the bulk of the labeled cells lie in sections A_7_–A_9_. The retrograde labeling (light purple) from the Un07 injection (P2−) is shown in Figure 6B, with the bulk of the labeling in sections B_8_–B_10_. Figure 6C depicts the retrograde cell labeling involving all injections in P2+lat and P2− collapsed onto a single series, showing that labeling from the P2+lat injection is caudal to that from the P2− injection. Note that the cells labeled from the P2− cells tended to lie ventral to that from the P2+lat injections (Figures 6C_8,9_).

### Analysis of Rostro-Caudal Differences of Retrograde Labeled Cell Distribution in the mcIO

**Figure 7:**
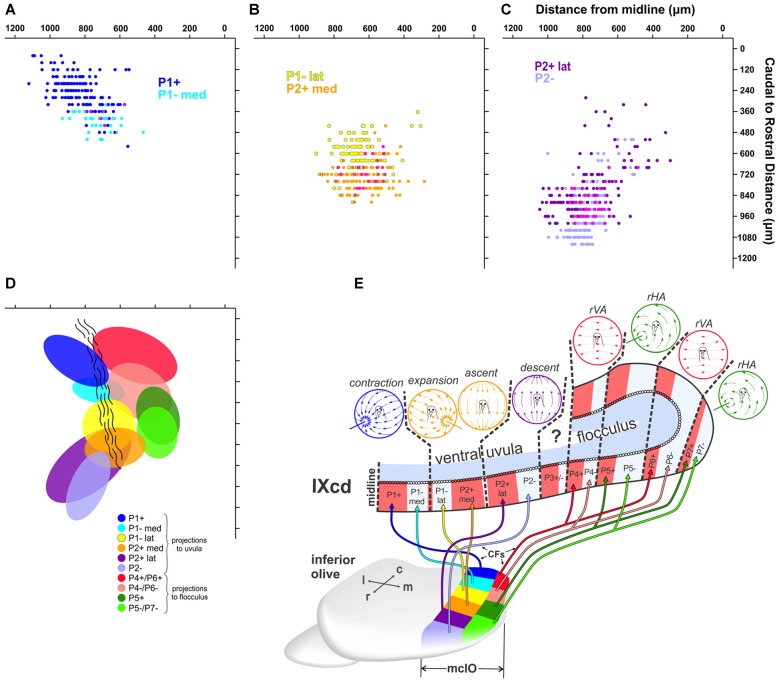
**(A–D)** Reconstructions of retrograde labeling in the mcIO as projected onto the horizontal plane. These were reconstructed from serial sections 40 μm apart in the rostro-caudal dimension (y-axes) and the distance of each retrogradely labeled cell from the midline was measured (x-axes). **(A)** shows retrograde labeled olive cells from injections in P1+ (dark blue; cases Uv01 green CTB injection, Uv12 red CTB injection; see Figure 3) and P1–med (light blue; Uv01 red CTB injection) stripes. **(B)** shows retrograde labeling from injections in P1–lat (yellow; Uv06 red, Uv08, Un05) and P2+med (orange; Uv06 green, Uv02, Uv16). **(C)** shows retrograde labeling from injections in P2+lat (dark purple; Uv11, Uv13) and P2– (light purple; Un07, Uv04). The magenta in **(A–C)** represents overlap. **(D)** shows 80% confidence ellipses that fit these six distributions. In addition, the regions that project to the ZII stripes in the flocculus (P4+/− to P7+/−) are shown based on data from Wylie et al. (2017). **(E)** shows a schematic of the climbing fiber projections (CFs) from each region of the mcIO to the ZII stripes in the optic flow zones in folium IXcd (Adapted from Pakan et al., 2014 with permission). c, caudal; r, rostral; m, medial; l, lateral.

The data illustrated in Figures 4–6 suggest that there are rostro-caudal differences in retrograde labeling from injections in each of the ZII+ and ZII− stripes in the uvula. Figure 7 shows quantitative support for this claim, where the location of retrograde labeled cells in mcIO are projected onto an horizontal plane for all injections except for the two injections that spanned both P1−med and P1−lat (i.e., green injections in Uv12 and Uv07; Figures 3B,C). The bulk of the labeling from the P1+stripe was located 700–1000 μm lateral to the midline and 100–300 μm from the caudal tip of mcIO. The labeling from the P1−med injection was rostral and slightly medial to this (350–450 μm from the caudal tip; 600–800 μm from the midline; Figure 7A). The bulk of the retrograde labeling from the P1−lat stripe was 500–750 μm from the midline and 500–650 μm from the caudal tip, whereas that from the P2+med injections was rostral to this, 700–800 μm from the caudal-tip (Figure 7B). Finally, shown in Figure 7C, the bulk of the labeling from the P2+lat injections was found 650–950 μm from the midline, 750–1000 μm from the caudal tip, whereas the labeling from the P2− stripe was slightly rostral to this, 900–1100 μm from the caudal tip. All of these data are summarized in Figure 7D, where 80% confidence ellipses were fit to these distributions using the *ggplot2* package in R (R Core Team, 2015). Included in this are the distributions of the mcIO cells projecting to the ZII stripes in the flocculus (P4+/− to P7+/−, from Wylie et al., 2017).

## Discussion

Studies of the pigeon VbC have previously shown that a ZII+/− stripe pair represents a functional unit insofar as the response of PC CSA to optic flow stimuli is consistent within a ZII+/− stripe pair (Pakan et al., 2011; Graham and Wylie, 2012; see Figure 7E). In the present study we showed that ZII+ and ZII− stripes within each functional unit in the uvula receive input from different, although adjacent areas of the mcIO. This is depicted in the schematic in Figure 7E. The projection to the uvula arises from the lateral mcIO, which can be divided into six rostro-caudal regions. The contraction zone receives input from the caudal-most lateral mcIO en masse, but the P1+ zone receives input from the most caudal end (dark blue), while the P1–med zone receives input from a slightly more rostral region (light blue), as well as slightly more medial region (Figures 4, 7A). The expansion/ascent zone is innervated by the middle lateral mcIO en masse, but the region projecting to the P1–lat stripe (yellow) is caudal to that projecting to the P2+med stripe (orange). Finally, the descent zone is innervated by the rostral lateral mcIO en masse, but the projections to P2+lat arises from a region of the mcIO (dark purple) that is slightly more caudal to that region innervating the P2− stripe (light purple). Note that the region innervating the P2− stripe is slightly ventral to that innervating the P2+lat stripe (Figures 6C_8,9_). Also included in the schematic in Figure 7E is the projection from the medial mcIO to the ZII stripes in the flocculus (Wylie et al., 2017). It is similar insofar as the input to the ZII− and ZII+ stripes of a functional zone arise from adjacent rostro-caudally separated regions in the mcIO. However, each of these regions innervates two stripes. For example, there are two VA zones in the flocculus, spanning stripes P4+/− and P6+/−. The most caudal region of the medial mcIO (red) innervates both the P4+ and P6+ stripes, whereas an area immediately rostral to this (pink) innervates the P4− and P6− stripes.

From work mostly in rodents, it is generally stated that that a particular olivary subnucleus innervates either ZII+ or ZII− stripes, but not both (Voogd et al., 2003; Sugihara and Shinoda, 2004; Voogd and Ruigrok, 2004; Pijpers et al., 2006; Sugihara and Quy, 2007). However, it is often adjacent subnuclei that project to adjacent ZII+ and ZII− stripes. For instance, Sugihara and Shinoda (2004) found that ZII+ stripes a+ and 2+ of the rat anterior vermis receive CF afferents from subnucleus c of the caudal medial accessory olive, while ZII− stripes a- and 2- receive input from the immediately adjacent subnucleus b. As olivary projections to ZII+ and ZII− regions arise from adjacent olivary nuclei in rodents, our scheme in which projections to different ZII stripe signatures arise from adjacent olivary regions is quite similar. It is possible that our six mcIO regions could exhibit different neurochemical properties and therefore be considered separate subnuclei (Vibulyaseck et al., 2015), but such divisions were not apparent in our thionine stained pigeon sections. Comparisons between the pigeon and mammalian IO must be approached with caution as the mammalian IO is complex and has more subdivisions than the avian IO. However, there are very clear comparisons that can be made between IO projections to the flocculus of mammals and pigeons. Floccular organization is strikingly similar in mammals and pigeons. As in pigeons, in rabbits, VA and HA zones interdigitate, and are innervated by separate olivary subnuclei (Voogd et al., 1996; Voogd and Wylie, 2004). In rats, rabbits and cats, the caudal dorsal cap of the principal olive projects to the VA zones and the ventral lateral outgrowth and the rostral dorsal cap to HA zones (for review see Voogd et al., 1996). As such, these two areas in the IO of mammals can been considered analogous to the pigeon medial caudal mcIO and dorsal mcIO respectively, particularly as they also receive similar optic flow information and project to interdigitating floccular zones (For review, see Voogd and Wylie, 2004). Comparing the pigeon uvula to the mammalian uvula is not as clear. In pigeons, the lateral caudal and rostral mcIO project to the lateral and medial uvula, respectively. In mammals, the β-nucleus and the dorsomedial cell column of the medial accessory olive project to the medial and lateral uvula, respectively. In this regard, these structures could be considered analogous in terms of their projections patterns. However, the mammalian and avian uvula differ in terms of their optic flow response property organization. PC CSA in the pigeon uvula responds to translational patterns of optic flow (Graham and Wylie, 2012), while in rabbits, PC CSA responds vestibular sensory information (Shojaku et al., 1991; Barmack and Shojaku, 1992, 1995). As such, the seemingly analogous olivary structures between pigeons and mammals process different types of sensory information, and so are not analogous in this regard.

Not only do the ZII+ and ZII− stripes within a functional unit receive different olivary inputs, there is also some evidence showing that they have functional differences, despite the fact the CSA responds best to the same pattern of optic flow. First, PCs in ZII+ stripes likely project to different areas within the cerebellar and vestibular nuclei than PCs in ZII− stripes (Sugihara, 2011; Wylie et al., 2012). Second, it is likely that mossy fibers project to ZII+ and ZII− stripes differentially. Pakan et al. (2010) showed that mossy fibers from two retinorecipient nuclei in pigeons, the nucleus of the basal optic root and the nucleus lentiformis mesencephali, generally terminated adjacent to ZII+ stripes in the VbC, including the ventral uvula. Therefore, ZII+ cells are likely receiving more visual input via the mossy fiber pathways. It is not known if mossy fiber inputs from other sensory systems project preferentially to ZII− stripes in the pigeon, but it is possible that ZII+ and ZII− stripes are processing different sensory information. In addition to visual inputs, the VbC receives secondary vestibular inputs (Pakan et al., 2008) as well as spinal inputs (Okado et al., 1987; Necker, 1994). It is possible that these preferentially target ZII− zones, but this remains to be seen. Finally, several studies in rodents have suggested that ZII+ and ZII− PCs differ with respect to the mechanism of synaptic plasticity during motor learning: ZII+ cells rely more on long term potentiation (LTP), whereas ZII− cells rely more on long term depression (LTD; Wadiche and Jahr, 2005; Paukert et al., 2010; Wang et al., 2011; Ebner et al., 2012; Hawkes, 2014; Zhou et al., 2014). The VbC is involved in the plasticity of the vestibulo-ocular response (VOR; du Lac et al., 1995), a reflex that is responsible for mediating retinal image stabilization. VOR enhancement is associated with LTD, while VOR suppression is associated with LTD (Broussard et al., 2011), so within a functional unit of the pigeon VbC, it is possible that the ZII+ stripe is primarily involved in VOR suppression while the ZII− stipes is involved in VOR enhancement. Thus, each functional unit consists of a stripe of ZII+ PCs and a stripe of ZII− that: (i) receive different CF inputs; (ii) receive mossy inputs from different sensory systems; (iii) project to different areas of the vestibular and cerebellar nuclei; and (iv) use LTP and LTD, respectively.

## Author Contributions

All authors had full access to all of the data in the manuscript and take responsibility for the integrity of the data and the accuracy of the data analysis. DRW and IC: conceived and designed the study. DRW, IC, JRC, CG-I and PLH: performed the experiments, acquired the data and data analysis. DRW, IC and CG-I: writing the manuscript. DRW: supervised the study.

## Conflict of Interest Statement

The authors declare that the research was conducted in the absence of any commercial or financial relationships that could be construed as a potential conflict of interest.
